# In Memoriam: David S. Cannom, MD—The Mirowski Fellowship and His Legacy in Japanese and Asia‐Pacific Electrophysiology

**DOI:** 10.1002/joa3.70505

**Published:** 2026-10-27

**Authors:** Itsuro Morishima

**Affiliations:** ^1^ Department of Cardiology Tono Chubu Medical Center Toki Japan

**Keywords:** Asia Pacific Heart Rhythm Society, David S. Cannom, implantable cardioverter‐defibrillator, Japanese Heart Rhythm Society, Mirowski fellowship

The international cardiac electrophysiology community lost one of its distinguished leaders and educators with the passing of David S. Cannom, MD, on August 9, 2026, at the age of 86. For the Japanese and Asia‐Pacific electrophysiology communities, Dr. Cannom was not only an internationally respected electrophysiologist but also a mentor and friend who played an important role in connecting generations of physicians with the international electrophysiology community (Figure 1).

**FIGURE 1:**
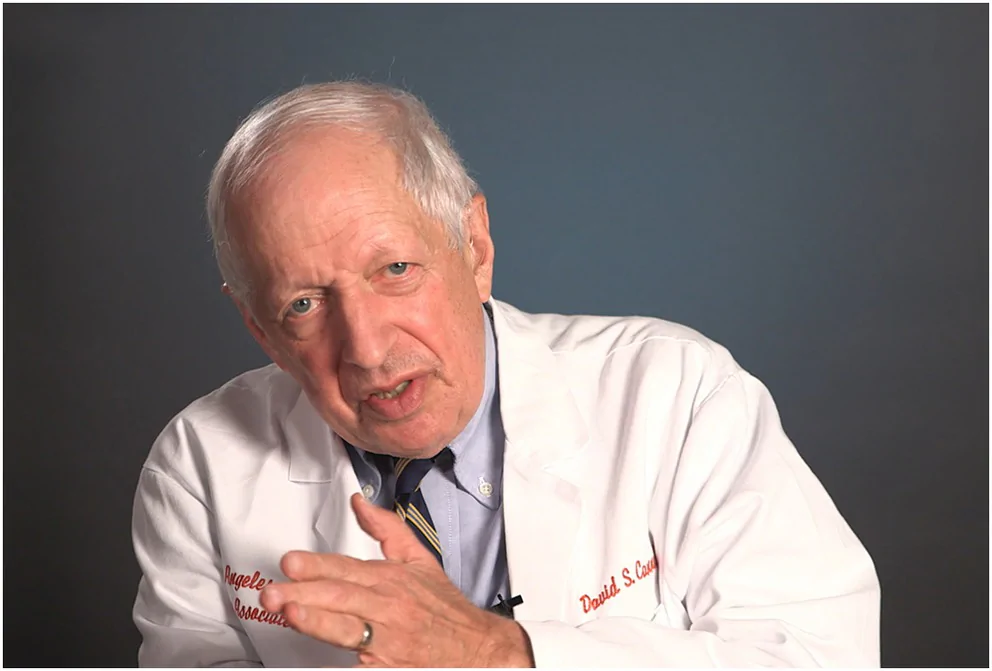
David S. Cannom, MD (1940–2026).

Dr. Cannom was one of the pioneers of modern clinical electrophysiology and the treatment of sudden cardiac death. During his long career at Good Samaritan Hospital in Los Angeles, he made major contributions to the advancement of implantable cardioverter‐defibrillator (ICD) therapy and the clinical management of life‐threatening ventricular arrhythmias. He also served as President of the North American Society of Pacing and Electrophysiology, now the Heart Rhythm Society.

One of his most enduring legacies was his commitment to education. From 1992 through 2014, a total of 57 Japanese physicians participated in the Mirowski Fellowship Program, organized by the Japanese Heart Rhythm Society (JHRS), and trained at Good Samaritan Hospital. The fellowship was named in honor of Dr. Michel Mirowski, who pioneered the development and first clinical application of the ICD. The names of these fellows are listed in Table S1. At a time when clinical electrophysiology was evolving rapidly, the program provided young Japanese physicians with a unique opportunity to experience state‐of‐the‐art electrophysiology in the United States. Dr. Cannom did not simply teach us electrophysiology; he made us feel that we belonged to the international electrophysiology community.

I was fortunate to participate in the Mirowski Fellowship Program in 2005. During my fellowship, Dr. Cannom was first and foremost my mentor. Over the years, however, our relationship gradually developed into a friendship, and I came to know him simply as David. I still vividly remember the first day I met him in Los Angeles. He told me, “I will let you be exposed to EP cases, device cases, conferences and lectures as best I can.” He fulfilled that promise completely. Through him, I was able to meet and learn from many distinguished electrophysiologists, including Dr. Koonlawee Nademanee, Dr. Kalyanam Shivkumar, and Dr. Eric Prystowsky, while also gaining broad exposure to the ideas, discussions, and perspectives that were shaping electrophysiology internationally. I learned from David far more than the technical aspects of electrophysiology. His clinical judgment and patient‐centered approach taught me that technical expertise in electrophysiology must always be grounded in careful clinical medicine.

His relationship with his fellows did not end when they returned to Japan. He continued to follow our careers and maintained a genuine interest in the development of electrophysiology in Japan. He visited Japan on many occasions in connection with meetings and activities of the JHRS, maintaining close contact with Japanese colleagues over many years. In 2013, he joined a reunion of former Mirowski Fellows in Tokyo (Figure 2). In 2016, he again joined former fellows at the Annual Meeting of the JHRS for the Mirowski Fellowship Program Memorial Lecture, reflecting the enduring relationship he had built with Japanese electrophysiologists.

**FIGURE 2:**
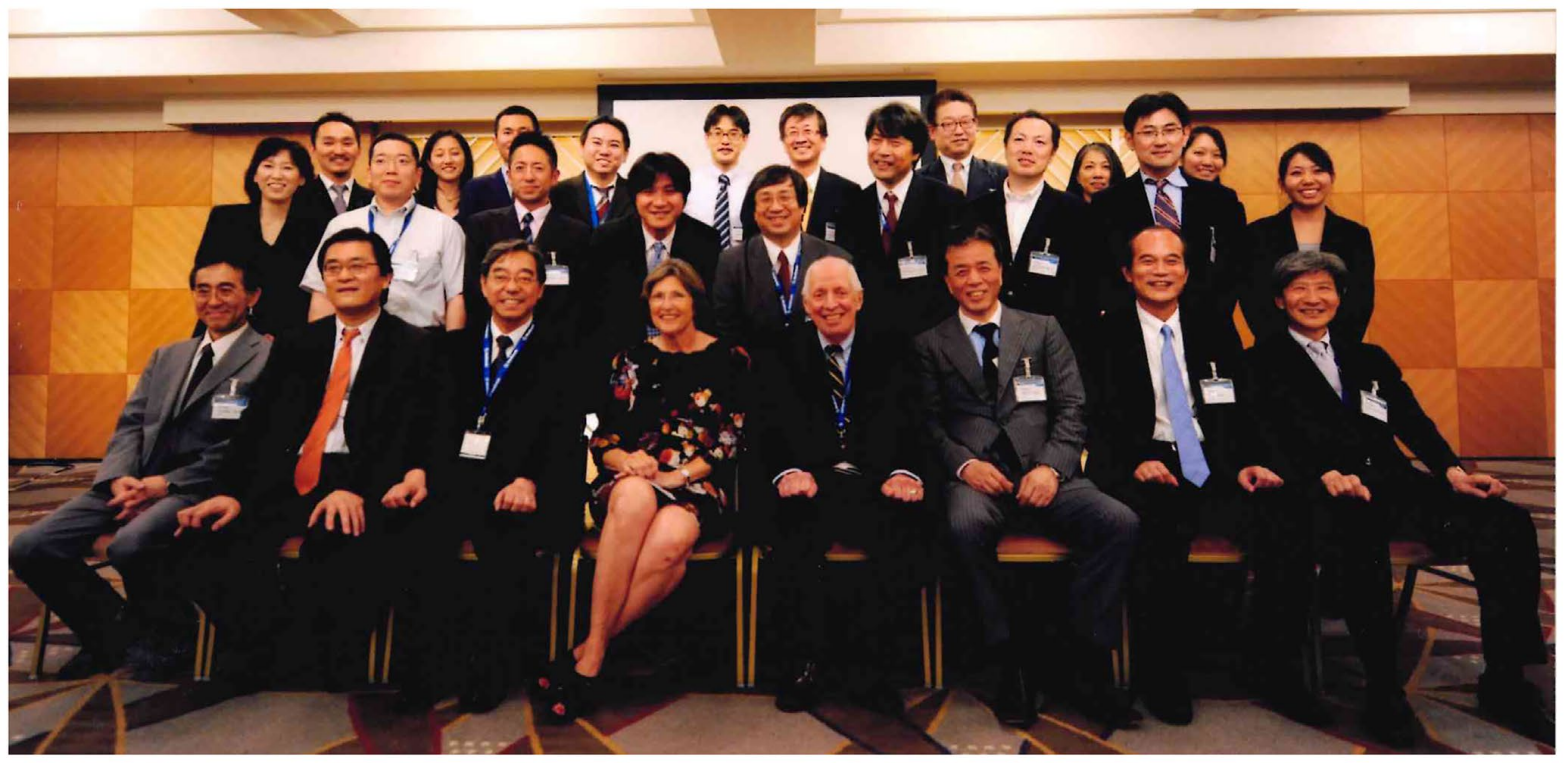
Reunion of former Mirowski Fellows, Tokyo, July 7, 2013. David S. Cannom and his wife, Phyllis, are seated at the center.

One of my most cherished memories of David dates from 2013, when he visited Nagoya with his wife, Phyllis. During two remarkably full days, he gave a lecture in Nagoya on the safety of sports participation for young patients with ICDs, visited Ogaki Municipal Hospital, where I was working at the time, and attended the Nagoya Grand Sumo Tournament with us. David was an avid sumo fan, and sharing the tournament with him and Phyllis was a particularly memorable experience. We also traveled to Gifu to see traditional cormorant fishing on the Nagara River. That evening, the river was flowing at approximately five times its usual speed. The fishing boats passed by rapidly, and we did not see a single ayu. David immediately turned the disappointment into humor, saying, “The fish had a good night because of the rapid currents and we were a good luck charm for them.” That comment captured his personality perfectly. He had a remarkable ability to find humor in almost any situation and to make those around him feel at ease.

David also participated in meetings of the Asia Pacific Heart Rhythm Society, where he engaged with physicians from across the Asia‐Pacific region and contributed to the exchange of knowledge and the development of lasting professional and personal connections. At the 2019 meeting in Bangkok, Dr. Yasushi Oginosawa, another Mirowski Fellow, and I had dinner with David and Phyllis. Afterward, as we said goodbye, I watched the two of them walking back to their hotel hand in hand. It was a simple but deeply memorable scene, and I remember thinking what a wonderful couple they were. That evening in Bangkok was the last time I saw David in person; the COVID‐19 pandemic that followed prevented us from meeting again.

Electrophysiology changed enormously during David's career, but one of his most enduring legacies is reflected in the physicians he trained and mentored. His influence continues in clinical practice, research, and education in Japan and across the Asia‐Pacific region. For those of us who had the privilege of learning from him through the Mirowski Fellowship Program, his passing is a profound loss. In expressing these thoughts, I believe I speak for many of the Japanese Mirowski Fellows who were fortunate to learn from him.

We will remember David with deep gratitude and affection for his mentorship, friendship, and enduring contribution to cardiac electrophysiology.

## Disclosure

The author has nothing to report.

## Conflicts of Interest

The author declares no conflicts of interest.

## Supporting information


**Table S1:** List of Mirowski fellows.

## Data Availability

The data that support the findings of this study are available on request from the corresponding author. The data are not publicly available due to privacy or ethical restrictions.

