# Supplementary material for: In Memoriam: David S. Cannom, MD—The Mirowski Fellowship and His Legacy in Japanese and Asia‐Pacific Electrophysiology
Source: J Arrhythm. 2026 Oct 27;42(6):e70505. doi: 10.1002/joa3.70505 (PMC13712318; doi:10.1002/joa3.70505)
Supplement: Supplementary file 1 [file JOA3-42-e70505-s001.docx]

**Supplementary Table 1. List of Mirowski Fellows**

.

**Year of Training	Fellows**

1992	Hiromi Ando; Shigeki Ito; Masataka Sumiyoshi; Izumi Yoshida

1994	Tsunesuke Kouno; Masaru Haga; Kei Miyoshi

1995	Haruhiko Abe; Masafumi Sueshiro; Yasuhisa Nishimoto

1996	Shuji Takahata; Yasuchika Takeishi; Hiroyuki Fujisaki

1997	Jun Suzuki; Akira Miyata

1998	Yoshihisa Enjoji; Joji Hosokawa; Naoki Matsumoto,

1999	Tamana Kanai; Yasushi Miyauchi; Masato Watanuki

2000	Yuji Okuyama; Hitoshi Suzuki; Kimiaki Chino

2001	Hiroki Sato

2002	Norihiko Ono; Toshihisa Nagatomo; Yukihiko Yoshida

2003	Kimie Okubo

2004	Yasushi Oginosawa; Shingo Sasaki

2005	Susumu Nakamoto; Itsuro Morishima

2006	Junji Kawagoe; Shinya Shimoshige

2007	Yuko Inoue; Masahiro Mizobuchi; Shonosuke Ryu

2008	Miki Yokokawa

2009	Mitsuharu Kawamura; Atsushi Suzuki

2010	Kanki Inoue; Eiichiro Nakagawa; Yoshiyuki Watanabe

2011	Hitoshi Minamiguchi; Yosuke Miwa

2012	Tomohito Inage; Yoshinari Enomoto; Yuko Hirao

2013	Shingen Owada; Ritsuko Kohno; Hirofumi Mitsuyama

2014	Daisuke Horiuchi; Takenori Yao; Yasuteru Yamauchi; Yoshihiro Yamashina; Atsuyuki Watanabe
